# Use of a beta microprobe system to measure arterial input function in PET via an arteriovenous shunt in rats

**DOI:** 10.1186/2191-219X-1-13

**Published:** 2011-08-10

**Authors:** Geoff Warnock, Mohamed-Ali Bahri, David Goblet, Fabrice Giacomelli, Christian Lemaire, Joel Aerts, Alain Seret, Xavier Langlois, Andre Luxen, Alain Plenevaux

**Affiliations:** 1 University of Liège, Cyclotron Research Center (B30), Allée du 6 Août, 8, 4000 Liège, Belgium; 2 Johnson and Johnson Pharmaceutical Research and Development, Turnhoutseweg 30, 2340 Beerse, Belgium; 3 Université de Liège, Imagerie médicale expérimentale, Institut de Physique B5, 4000 Liège, Belgium

**Keywords:** beta microprobe, arterial input function, PET, rat

## Abstract

**Background:**

Kinetic modeling of physiological function using imaging techniques requires the accurate measurement of the time-activity curve of the tracer in plasma, known as the arterial input function (IF). The measurement of IF can be achieved through manual blood sampling, the use of small counting systems such as beta microprobes, or by derivation from PET images. Previous studies using beta microprobe systems to continuously measure IF have suffered from high background counts.

**Methods:**

In the present study, a light-insensitive beta microprobe with a temporal resolution of up to 1 s was used in combination with a pump-driven femoral arteriovenous shunt to measure IF in rats. The shunt apparatus was designed such that the placement of the beta microprobe was highly reproducible. The probe-derived IF was compared to that obtained from manual sampling at 5-s intervals and IF derived from a left ventricle VOI in a dynamic PET image of the heart.

**Results:**

Probe-derived IFs were very well matched to that obtained by "gold standard" manual blood sampling, but with an increased temporal resolution of up to 1 s. The area under the curve (AUC) ratio between probe- and manually derived IFs was 1.07 ± 0.05 with a coefficient of variation of 0.04. However, image-derived IFs were significantly underestimated compared to the manually sampled IFs, with an AUC ratio of 0.76 ± 0.24 with a coefficient of variation of 0.32.

**Conclusions:**

IF derived from the beta microprobe accurately represented the IF as measured by blood sampling, was reproducible, and was more accurate than an image-derived technique. The use of the shunt removed problems of tissue-background activity, and the use of a light-tight probe with minimal gamma sensitivity refined the system. The probe/shunt apparatus can be used in both microprobe and PET studies.

## Background

With the increased popularity of positron emission tomography (PET) imaging in small animals, the use of kinetic modeling to study physiological function has become widespread. Many kinetic models, for example those used in the quantitative measurement of local cerebral glucose metabolism or measurement of radiotracer receptor binding, require the accurate measurement of the time-activity curve of the tracer in plasma, known as the input function (IF). In the case of the widely used tracer ^18^F-fluorodeoxyglucose (FDG), the time-activity curve of tracer uptake in a specific brain region, combined with the IF, can be used in a classical modeling approach to calculate the kinetic rate constants governing passage from one compartment to another, and to calculate the rate of glucose metabolism. In the case of tracers used to image neurotransmitter receptors in the brain, an accurate IF facilitates kinetic modeling avoiding the assumptions of reference region models, namely that the reference region is devoid of the receptors studied and that the non-specific distribution volume is identical between reference and target region.

A number of methods have been described for the determination of IF in small animals. Manual blood sampling techniques have been frequently used to estimate IF. Indeed these techniques are considered as the gold standard for IF determination, despite the fact that these methods can limit the temporal resolution between measurements, and through the reduction of blood volume, may influence the animal's physiology [1]. Some groups have successfully implemented micro-blood sampling techniques to reduce the impact of these problems [2,3]. Blood counter systems have also been described for both humans and animals [4-8] which allow continuous counting in a flowing catheter to achieve high time resolution. Beta microprobe systems have also been used to continuously measure IF without the need for blood sampling, in humans, primates, and rodents [9-13]. Non-invasive techniques using a PET scan of the heart to determine IF in small animals have also been developed [10,14-23]. These methods use either a volume-of-interest (VOI) drawn in the left ventricle or mathematical analysis to extract the IF from the image. The use of a standardized IF between animals, calibrated by one or two blood samples [24,25] and a combination of image-derived and blood sampling methods have also been described [26,27].

Here, we describe the use of a beta microprobe to continuously measure IF in rats with minimal dead volume and without the need to subtract background tissue activity, which was a problem in previous studies using microprobes [12,13]. To achieve this, an arteriovenous shunt was placed between the femoral artery and vein, in a manner similar to that described by Weber et al. [28]. Similar shunt systems have also been reported previously [29,30]. However, in contrast to the method described by Weber et al. [28], a microprobe was placed directly into the shunt blood flow instead of passing the catheter through a separate coincidence counter. Pain et al. [12] placed a microprobe directly into the femoral artery to measure IF, which removes the need for a shunt and the potential of extra cardiovascular load, but due to the accumulation of tracer in adjacent tissues the use of a second probe to subtract background activity was required. This problem is also described by Laforest et al. [10]. Seki et al. [13] also used a two-probe shunt system to measure IF in rhesus monkeys, with one probe used specifically to subtract gamma radiation from the overall (beta/gamma) signal. In the present study, a single probe (Swisstrace, Switzerland) was used, and the shunt apparatus designed such that its placement was highly reproducible. A further advantage of the Swisstrace probe used is the light-impermeable coating. As the probe itself is insensitive to light, it is possible to use clear materials in the shunt, allowing visualization of the probe and confirmation of correct positioning.

In the present study, the IF derived using the beta microprobe shunt apparatus was compared to manual blood sampling at 5-s intervals during the peak phase, and to the IF derived from a left ventricle VOI in a dynamic PET image of the heart to confirm its accuracy.

## Materials and methods

### Animals

Male OFA (Oncins France souche A - Sprague Dawley) rats were initially obtained at 5 weeks of age from Charles River Laboratories (Bruxelles, Belgium) and subsequently bred at the Animal Facility of the GIGA-University of Liege (BE-LA 2610359; Liege, Belgium). Mean (± SD) body weight at testing was 273 ± 46 g. The animals were housed under standard 12 h:12 h light/dark conditions with food and water available *ad libitum*. All experimental procedures and protocols used in this investigation were reviewed and approved by the Institutional Animal Care and Use Committee of the University of Liege.

### Beta microprobe system

A commercially available beta microprobe system [31] (Swisstrace, Zurich, Switzerland) was used for the measurement of radioactivity in the blood. PMOD software version 2.95 (PMOD Technologies Ltd., Zurich, Switzerland) was used for the acquisition of data from the photomultiplier tubes. The system allows counting of radioactivity with a temporal resolution of 1 s. The physical characteristics of the system and probes have been described elsewhere [31]. The linearity of the system was confirmed by measuring counts from a series of solutions of known activity spanning a wide range to encompass the range of experimental values.

### Shunt apparatus and surgery

The animals were anesthetized using isoflurane in 30% oxygen/70% nitrous oxide, and polyethylene catheters (PE20; prefilled with 50 U/ml heparinized saline) were implanted in femoral artery and vein. The catheters were connected to an arteriovenous shunt driven by a peristaltic pump (Watson-Marlow 403U/R1, with 0.8 ID × 1.6 OD mm Pumpsil tubing, Wilmington, MA, USA) at a standard flow rate of 28.60 ± 0.18 ml/h (mean ± SD; at room pressure) in agreement with that reported by Weber et al. [28]. The pump was activated 5-10 min before PET measurements. T-connections in the shunt allowed monitoring of blood pressure via a pressure transducer, intravenous infusion and the insertion of a probe tip into the blood. The arterial catheter was shortened such that the distance between artery and probe was 10-15 cm. Distal to the probe the shunt was 37 cm in length (including the peristaltic pump tubing) and the venous catheter was also shortened to a length of 10-15 cm. For the collection of blood samples for manual counting, a second arterial catheter was implanted in the second femoral artery.

**Figure 1:**
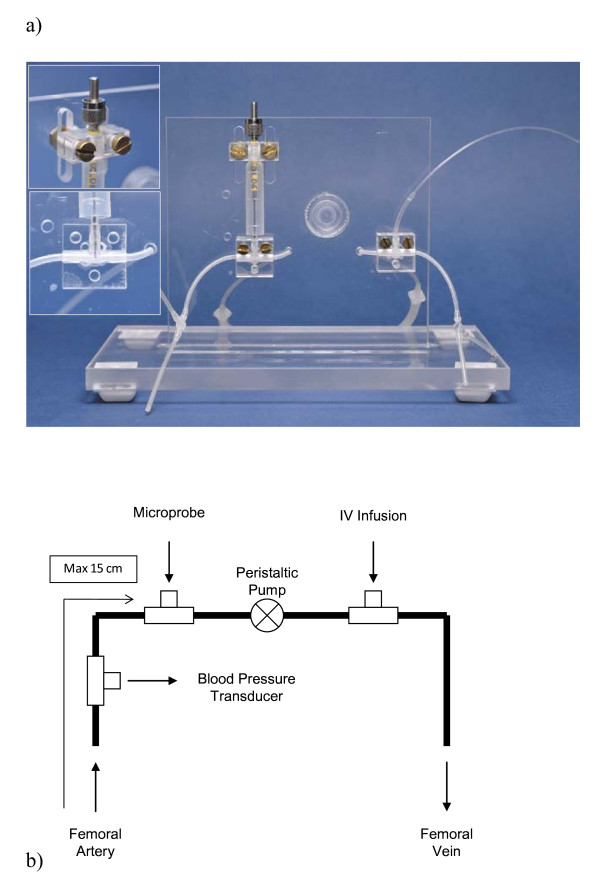
**The shunt apparatus**. (**a**) The shunt apparatus mounted on the acrylic stand with adjustable clamps (upper probe mount and tip location inlaid); (**b**) Diagram illustrating the design of the shunt, with T-connections for blood pressure measurement, probe insertion, and intravenous infusion. The peristaltic pump is connected after the probe in series in order to prevent backflow from the IV infusion connection affecting probe measurements.

The shunt apparatus consisted of silicone tubing (0.040" ID × 0.085" OD Silclear tubing, Degania Silicone, VWR International, Benelux) mounted on a Plexiglas stand with secure mounts for the T-connections (Harvard Apparatus Standard tube T-connector #72-9275 1.5 mm ID, Harvard University, Cambridge, MA, USA) and a secure, adjustable, mounting point for a microprobe, as illustrated in Figure 1. Plexiglas was chosen for the stand as it could readily be cleaned and would not corrode after contact with saline. The T-connections and microprobe are clamped in position with custom machined Plexiglas clamps, such that the apparatus can readily be dismantled for cleaning or replacement of parts but with precise reassembly. The clamp for the microprobe clamps at the solid base of the probe and is height adjustable via two machined slots to account for differences in probe lengths. The shunt was prefilled with 250 U/ml heparinized saline (volume approximately 400 μl). The probe connection was situated such that arterial blood first passed over the probe tip for counting, followed by the peristaltic pump, to prevent back-flow when infusing via the second T-connection. Finally, the blood was pumped back into the animal via the femoral vein. A probe was fixed with its tip in the blood flow of the shunt for the measurement of IF. In this position, the probe has a measured sensitivity of 0.045 cps/kBq/ml (4.5%). The sensitivity of the probe is a function of crystal size at the measuring tip and detection volume. The detection volume is dependent on the energy of the radioisotope used and is approximately spherical around the probe tip [32]. In the shunt apparatus, the detection volume is partially filled with blood. Utilization of the full detection volume would require larger T-connections and therefore substantially increase the dead volume.

For calibration of the shunt probe immediately after IF measurement, the shunt was first flushed with distilled water, followed by a solution of known radioactivity. The activity of this solution was selected such that the counts recorded were in the same range as the experimental values. The position of the probe was precisely maintained between IF measurement and calibration. In this way, the system was calibrated in precisely the same position as used for IF acquisition. The reproducibility of the probe location in the apparatus was assessed by comparing the calibration factors calculated between individual studies. Cleaning and maintenance of the shunt between studies was performed with an enzymatic cleaning solution (Enzol, Advanced Sterilization Products, Irvine, CA, USA) and sterilizing solution (Cidex OPA, Advanced Sterilization Products, Gargrave, UK).

### Comparison of probe-derived, image-derived and manually sampled input functions

To simultaneously compare the IF determined by either microprobe, PET or manual sampling, Glucotrace (^18^F-fluorodeoxyglucose; FDG; 144.4 ± 10.7 MBq, Best Medical, Belgium) was injected as an intravenous bolus (0.5-0.6 ml over 30 s) in eight rats, via the T-connection on the intravenous side of the shunt apparatus. The animals were not fasted prior to the study. Activity in the blood was counted using the microprobe system, while blood samples (100-150 μl) were collected every 5 s for 90 s, then at 120, 180, 240, 300, and 600 s after FDG infusion from the second arterial catheter. The samples were collected in 1 ml of heparinized saline for manual counting of the whole blood activity in a calibrated gamma spectrometer (high purity 30% germanium GR3020 Canberra Industries, Meriden, CT, USA).

Cardiac IF was simultaneously derived from dynamic PET images in the same eight rats using a Siemens Focus 120 microPET scanner (Siemens, Munich, Germany). The physical characteristics of this scanner have been described elsewhere [33]. The heart was placed in the center of the scanner field-of-view.

PET studies started with a 10-min transmission scan carried out using a ^57^Co point source with single event acquisition mode using a 120-125-keV energy window. FDG was injected following the transmission scan. Emission data was then recorded in list mode for a total of 30 min. Emission data was acquired with an energy window of 350-650 keV and a coincidence-timing window of 6 ns. Data was reframed with a high temporal resolution, especially for the first 3 min, in order to accurately delineate peak activity in the blood (30 × 2 s, 10 × 10 s, followed by 30-s frames). Images were reconstructed with all corrections using Fourier rebinning and filtered backprojection with a ramp filter cutoff at the Nyquist frequency. A total of 95 transaxial slices were obtained in a 256 × 256 matrix. The slice thickness was 0.796 mm, and the in-slice pixel size was 0.433 mm. To avoid spillover effects, a spherical volume-of-interest (VOI) of 2 mm diameter was drawn in the left ventricle of the heart for derivation of the IF. This VOI diameter was selected after trials with varying diameters. Two millimeters was selected as the best compromise between increased noise in smaller VOIs and obvious spillover (increasing counts in the tail of the IF) in larger VOIs. The positioning of the 2 mm VOI can be seen in Supplementary Figure 1 of see Additional file 1.

In addition to visual comparison, the area under the curve (AUC) determined via the three methods was compared as a measure of the similarity between IF obtained via either probe, PET or manual sampling. AUC was determined for the first 10 min of data. For AUC calculations, the probe-derived IF was adjusted for delay and resampled with 2-s initial frames to match the PET data (in separate studies the delay introduced by the shunt apparatus has been measured at 10.3 ± 3.0 s (mean ± SD; n = 7) using fitting in PMOD software; using brain time-activity curves and probe-derived IFs for another tracer, the delay in the IF was fitted as part of a two-compartment kinetic model). To reduce the effect of noise on the AUC calculations, all IFs were fitted using a bi-exponential model in PMOD and AUC was also compared between the fitted IF curves. Using the values from the fitted bi-exponential model the slopes of the IFs derived from the three methods were compared for further evaluation of the similarity between the IFs.

### Influence of shunt length, pump speed and arterial source

The influence of shunt length (and thus volume), flow rate by altering the speed of the peristaltic pump and the influence of arterial source (i.e., femoral artery or carotid artery) were investigated in separate studies (*n *= 2/study/condition). The length of the arteriovenous shunt was increased by using longer PE20 catheters from femoral artery and vein (such that the distance from artery to probe was 45 cm compared to the usual 15 cm). Furthermore, a "minimum-possible-volume" shunt was connected between carotid artery and jugular vein, using a modified apparatus with a single T-connector for probe insertion (such that the distance from artery to probe was approximately 5 cm and the total shunt length 10-15 cm). The effect of flow rate was investigated by altering the speed of the peristaltic pump, using pump speeds of 14.30 ± 0.31, 28.60 ± 0.18, or 57.20 ± 0.63 ml/h (mean ± SD). A double injection protocol was used. "Standard" flow rate (28.60 ± 0.18 ml/h) was maintained during the first FDG uptake period (1 h), after which the flow rate was changed and a second dose of FDG administered. Consecutive FDG injections were separated and corrected for residual activity from the first injection using a two-exponential model in PMOD. The curves were aligned for the start of the IF peak, and corrected for injected activity. To study the effect of arterial source an arteriovenous shunt was connected between carotid artery and femoral vein, for comparison to the femoral-femoral situation (the distance from the carotid catheter site to the probe was approximately 15 cm, comparable to the femoral situation).

### Statistics

The influence of IF-derivation method on AUC from non-fitted and fitted IFs was compared using ANOVA followed by Student's *t *test for post hoc comparisons.

## Results

The mean sensitivity calculated for the probe located in the arteriovenous shunt was 0.0452 ± 0.002 cps/kBq/ml (*n *= 8), with a coefficient of variation of 0.04. This is comparable to the mean sensitivity calculated from ongoing studies in our lab (*n *> 20), which was 0.0448 ± 0.007 cps/kBq/ml, with a coefficient of variation of 0.17.

### Comparison of probe-derived, image-derived, and manually sampled input functions

**Figure 2:**
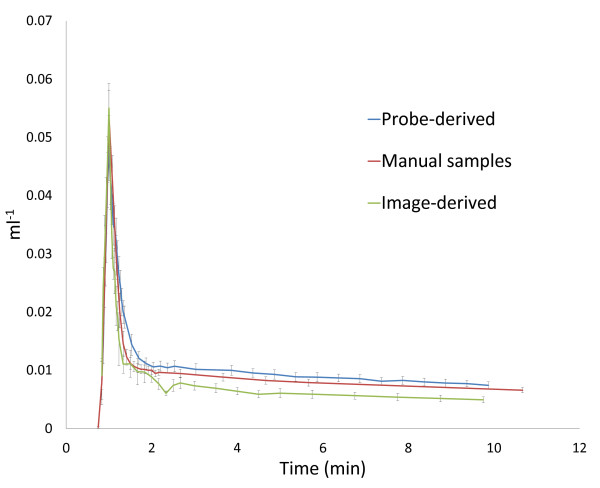
**Mean arterial input functions derived from the three methodologies**. Individual input functions were corrected for injected activity and aligned by the point of peak activity. Individual data points represent mean ± SEM. (Figures displaying the individual input functions in overlay are available as supplementary data).

A rapid peak in blood activity was recorded with the beta microprobe, which steadily fell until cessation of recording (mean curve corrected for injected activity, compared to the manually sampled and image-derived mean IF is shown in Figure 2; see Supplementary Figure 2 of Additional file 2 for overlaid figures displaying the reproducibility between individual IFs). A high degree of matching was seen between probe-derived IFs and corresponding manual blood samples. No notable dispersion was observed. The AUC for all IFs is compared in Table 1. The ratio of probe-derived to manual sampled AUC was 1.07 ± 0.05 (mean ± SD), with a coefficient of variation of 0.04. After fitting of the IFs to remove noise, the ratio of probe-derived to manual sampled AUC was 1.06 ± 0.05 (mean ± SD), with a coefficient of variation of 0.05. Statistical analysis revealed no significant difference in AUC between the probe-derived and manual sampled IFs (non-fitted: ANOVA *F*_2,21 _= 3.67, *p *= 0.043; *t *test *t*_(14) _= 0.52, *p *= 0.61. Fitted: ANOVA *F*_2,21 _= 4.3551, *p *= 0.026; *t *test *t*_(14) _= -0.46, *p *= 0.65). In contrast, image-derived IFs were significantly underestimated (mean IF corrected for injected activity shown in Figure 2) compared to either manual sampled or probe-derived IFs. The ratio of image-derived to manual sampled AUC was 0.76 ± 0.24 (mean ± SD), with a coefficient of variation of 0.32. After fitting of the IFs to remove noise, the ratio of image-derived to manual sampled AUC was 0.73 ± 0.21 (mean ± SD), with a coefficient of variation of 0.29. Statistical analysis revealed that the underestimation of the IF reached significance in the fitted data with only a trend in the non-fitted data (non-fitted: *t *test *t*_(14) _= 2.07, *p *= 0.057. Fitted: *t *test *t*_(14) _= 2.34, *p *= 0.035). The ratio of probe-derived to image-derived AUC was 1.52 ± 0.43 (mean ± SD), with a coefficient of variation of 0.28. After fitting of the IFs to remove noise, the ratio of probe-derived to image-derived AUC was 1.56 ± 0.41 (mean ± SD), with a coefficient of variation of 0.26. Slope constants for the two-exponential components of the fitted IF models are compared in Table 2. Statistical analysis revealed significant differences (ANOVA *F*_2,21 _= 19.44, *p *< 0.001) between manual- and probe-derived (*t *test *t*_(14) _= 2.37, *p *= 0.033) and manual- and image-derived (*t *test *t*_(14) _= -3.48, *p *= 0.003) IFs in the slope of the first exponential (slope 1;). However, no significant difference was found between manual- and probe-derived IFs for the slope of the second exponential (slope 2; ANOVA *F*_2,21 _= 12.54, *p *< 0.001; *t *test *t*_(14) _= -1.14, *p *= 0.27), which has a greater influence on the shape of the IF. In contrast, there was a significant difference in slope 2 between manual- and image-derived methods (*t *test *t*_(14) _= -4.13, *p *= 0.001).

**Table 1:** Area under the curve ratio between probe-derived, image-derived and manually sampled input functions

	Subject										
	1	2	3	4	5	6	7	8			
	AUC	AUC	AUC	AUC	AUC	AUC	AUC	AUC	Mean	SD	COV
Unfitted IF											
Probe IF	21,173	27,576	19,765	19,024	23,710	17,389	33,630	19,448			
Manual IF	20,246	26,385	18,418	17,265	22,878	15,270	30,684	19,538			
Image-derived IF	13,283	27,099	9,486	19,543	14,565	14,563	16,506	12,149			
											
Probe/manual	1.046	1.045	1.073	1.102	1.036	1.139	1.096	0.995	1.067	0.045	0.042
Pet/manual	0.656	1.027	0.515	1.132	0.637	0.954	0.538	0.622	0.760	0.240	0.315
Probe/PET	1.594	1.018	2.084	0.973	1.628	1.194	2.037	1.601	1.516	0.425	0.281
											
Fitted IF											
Probe IF	21,720	27,762	19,866	18,595	23,573	17,347	33,434	19,344			
Manual IF	20,981	26,414	18,500	17,602	23,402	15,226	30,297	19,693			
Image-derived IF	13,533	26,243	9,094.8	19,158	14,793	11,393	16,937	12,574			
											
Probe/manual	1.035	1.051	1.074	1.056	1.007	1.139	1.104	0.982	1.056	0.050	0.048
Pet/manual	0.645	0.994	0.492	1.088	0.632	0.748	0.559	0.639	0.725	0.210	0.290
Probe/PET	1.605	1.058	2.184	0.971	1.594	1.523	1.974	1.538	1.556	0.408	0.262

AUC, area under curve; COV, coefficient of variation; IF, input function; SD, standard deviation.

**Table 2:** Bi-exponential fit slope constants for probe-derived, image-derived and manually sampled input functions

	Subject								Mean	SD
	1	2	3	4	5	6	7	8		
Slope 1										
Probe IF	4.609	4.181	3.646	3.470	4.989	3.552	3.181	3.658	3.911	0.582
Manual IF	7.627	4.847	3.646	7.746	5.582	6.038	3.215	4.591	5.411	1.570
Image-derived IF	11.626	7.983	8.705	10.502	9.327	6.343	5.888	8.149	8.565	1.815
Slope 2										
Probe IF	0.048	0.038	0.046	0.028	0.047	0.025	0.039	0.038	0.039	0.008
Manual IF	0.042	0.018	0.050	0.043	0.039	0.031	0.008	0.026	0.032	0.013
Image-derived IF	0.095	0.068	0.051	0.056	0.082	0.040	0.059	0.072	0.065	0.017

IF, input function; SD, standard deviation.

### Influence of shunt length, flow rate, and arterial source

**Figure 3:**
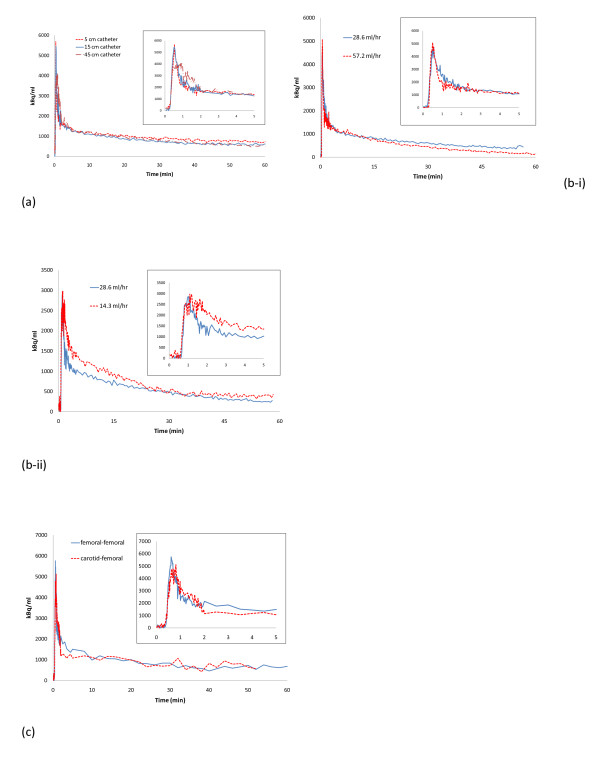
**Shunt length, flow rate, and arterial source**. (**a**) Effect of shunt length on dispersion of the input function peak (first 5 min shown). Data aligned for start of peak and corrected for injected activity for comparison purposes. (**b**) Effect of shunt flow rate on input function shape. (i) Effect of increasing pump speed (inset: first 5 min); (ii) Effect of reducing pump speed (inset: first 5 min). Data aligned for start of peak and injected activity for comparison urposes. (**c**) Comparison of input functions derived from femoral artery-femoral vein shunt to carotid artery-femoral vein shunt (first 5 min shown). Data from two individual subjects. Data aligned for start of peak and corrected for injected activity for comparison purposes.

Substantially increasing the length of the shunt (from 15 to 45 cm between artery and probe) had a clear dispersing effect of the peak of the IF (Figure 3a). In contrast, shortening of the shunt (from 15 to 5 cm between artery and probe) appeared to have no clear effect (Figure 3a). While doubling of the shunt flow rate appeared to have no detectable influence on the shape of the IF, as shown in Figure 3b-i, a halving of the flow rate was sufficient to produce dispersion in the IF (Figure 3b-ii). The shape of the IF did not appear to be affected by the choice of artery for catheter implantation (Figure 3c).

## Discussion

In the present study, we report the use of a specially designed arteriovenous shunt apparatus for use in combination with a beta microprobe system to determine input function (IF) in rats. This apparatus can be used in studies of radioligand accumulation or binding in small animals using either beta microprobe systems or microPET. The major advantages of our system are very high temporal resolution and no blood loss, while its major disadvantages are its invasive nature and low sensitivity.

The probe-derived IF closely matched that derived by manual blood sampling, the so called "gold standard", as shown by visual comparison (Figure 2) and in the comparison between areas under the curve (AUC) (Table 1). The difference in AUC observed may be attributable to statistical noise but also the difference in temporal resolution between these techniques. Although blood samples were taken with a resolution of 5 s, the probe system has a maximum resolution of 1 s. In contrast to the high degree of agreement shown between probe-derived and manual sampling derived IFs, image-derived IFs showed an underestimation and increased variability (Figure 2 and Table 1). PET-derived IF based on a volume-of-interest in the left ventricle of the heart has been described for use as a true arterial IF in a number of species [10,16,17,19-21]. Fitting of the IFs to reduce noise had a negligible effect on the coefficient of variation for the AUC ratios with all methods (Table 1). In addition to AUC, the slope constants of a bi-exponential model fitted to the IF curves were compared. Although AUC is a useful method for comparing the integrated activity present in the blood over the duration of the study, a drawback of the method is that it is possible for curves with very different shapes to have similar AUCs. In the present, study it was shown that the shapes of the IF curves are very similar (Figure 2), but comparison of the slope constants of the bi-exponential fit provides an indication of the instantaneous level of radioactivity in the blood, which is of importance for kinetic modeling using the IF curves. Statistical analysis revealed that all methods differed for the first slope constant, but that there was no significant difference between the probe- and manually derived curves in the second slope constant (Table 2). Changes in the first slope constant have a limited effect on the shape of the input function, while the second constant governs the decline in activity after the peak. Thus, the similarity in the second slope constant between probe- and manually derived curves supports the result of the AUC comparison and the conclusion that these IF curves are well matched.

A partial-volume effect is likely to be involved in the difference between image-derived IF and the gold standard or probe-derived IF. It is well documented that partial-volume effects adversely affect quantitative measures from small VOIs less than two times the FWHM resolution. The Siemens Concorde Focus 120 has a measured resolution of 1.2-1.5 mm [34,35], while the VOI used for IF determination had a diameter of 2 mm to avoid spillover. It is thus likely that a partial-volume effect leads to underestimation of the activity in the blood. Furthermore, spillover of activity from the myocardium is a serious problem in cardiac ventricular image-derived IF in rats and mice. In the present study in rats, spillover was reduced by using a small VOI located centrally in the left ventricle. While spillover can be corrected for mathematically [36,37], a clear advantage of the probe-derived IF is the lack of spillover. Methods combining image-derived IF and later blood sampling may also help minimize spillover effects [26,27]. Factor analysis of cardiac PET images can be used to separate the ventricular and myocardial components of the image [14,15,17-19,21-23], though calibration of the IF obtained to a blood sample may still be necessary.

A further drawback of PET-derived IF in microPET studies of the brain is the need to position both the heart and region of interest (e.g., cerebral cortex) in the field-of-view. Particularly where regions of interest are of the same scale as individual voxels, such as in the brain, it is well known that positioning that organ centrally in the field-of-view offers optimal spatial resolution. In order to position the brain and heart in the field-of-view of small animal scanners such as the Focus 120 simultaneously, it is necessary to position both organs at or near the extremes of the field-of-view. This may be detrimental to the overall data. Even in the human situation, where scanner resolution is less critical, a reliable method for image-derived input function without some degree of blood sampling has not yet been achieved [38].

Dispersion of the probe-derived IF due to the catheters and the shunt, and sticking of the tracer on the catheter walls are potential drawbacks of using an arteriovenous shunt. It has been shown that increasing catheter length and decreasing pumping speed increase dispersion in catheter-based blood detectors [7], a conclusion supported by the data in the present study (Figure 3a, b). Based on calculations for dispersion in the catheter prior to detection [39], Convert et al. [7] reported that correction of dispersion should be unnecessary for PE10 catheters where flow rate is greater than 125 μl/min (7.5 ml/h) or a length < 10 cm, or for PE50 catheters with flow rate > 250 μl/min (15 ml/h) or length < 20 cm. In the present study, PE20 catheters are used with a length < 20 cm and a measured flow rate equivalent to 477 ± 25 μl/min (15 ml/h). Thus, the effect of dispersion should be minimal, as supported by the similarity between probe-, image-, and manually derived IFs in the present study. Therefore, any dispersion effect can thus be minimized by keeping both catheters and shunt tubing to their minimum lengths, minimizing the total volume, and by maintaining a sufficient flow rate. If the circumstances of a given study do not allow optimization of these parameters, methods for the correction of dispersion have been described [40-42]. The carotid artery could also be used to obtain a probe-derived IF curve and minimize the distance from the heart to reduce dispersion. However, in the case of studies of brain function, it may be desirable to avoid possible changes in blood supply to the brain due to catheterization of the carotid artery. Furthermore, the comparison of carotid- and femoral-derived IFs in the present study indicated negligible differences (Figure 3c). The influence of a large volume shunt in the present study was clear (Figure 3a), while the difference between an extra-short shunt and a longer pump-driven shunt was minimal (see also Figure 3a), suggesting that only large increases in overall volume are significant. As above, a low pump speed is undesirable as this can increase dispersion. Indeed, halving the flow rate in the present study introduced a dispersion effect (Figure 3b). Including a pump in the shunt could be considered useful to standardize the flow rate between animals. Adsorption of the tracer can be accounted for by calibrating the shunt using a solution of known radioactivity pumped through the shunt with probe *in situ*, as described in the Materials and methods section. Thus, the calibration factor for the shunt probe will also be affected by tracer adsorption.

Reproducible positioning of the probe was facilitated by the shunt design (see Figure 1), and was confirmed by comparing the calculated sensitivity values. The calculated coefficient of variation was 0.04. This reproducible positioning also ensures that the minimal sensitivity of the Swisstrace probe (Swisstrace, Zurich, Switzerland) to Cerenkov radiation [31] is also unlikely to be of consequence to the accuracy of the measured IF, as the number of counts attributable to Cerenkov radiation was concluded to be related to the depth of the scintillating fiber in the volume of activity. In the shunt-probe apparatus, the depth of probe insertion into the blood flow is limited to approximately 1 mm, and the design allows this depth to be highly reproducible. It has been argued that beta microprobe systems can be difficult to use [10], particularly as many systems are highly sensitive to ambient light. The light-tight coating of the Swisstrace microprobes eliminates any influence of ambient light and removes this technical difficulty.

Recently, a method for microfluidic blood sampling has been described for IF measurement in small animals [3]. Although this elegant approach solves a number of difficulties with blood sampling in small animals, it is still not possible to achieve the time resolution of the beta microprobe-derived IF or avoid blood loss entirely. The removal of blood is, however, often required in studies with novel radiotracers, for the determination of metabolites present in the plasma. In this case, the use of an arteriovenous shunt is no more invasive than the use of a catheter solely for blood sampling. Methods using blood sampling to determine IF may have sufficient volume to measure metabolites directly. While the present arteriovenous shunt method allows the investigator to avoid blood loss where desired, the inclusion of a T-connection for blood withdrawal facilitates sampling for metabolites. Furthermore, intravenous tracer and drug administration is also facilitated.

Coincidence counters have also been used to measure activity in flowing catheters [7,28] with high time resolution. Due to the increased sensitivity of these counters, a lower dose of radiotracer can be used, which represents an advantage over the probe-derived system. At present, the availability and price of miniaturized coincidence counters represent disadvantages compared to beta microprobes. Furthermore, adequate shielding is required for these counters which could necessitate longer catheters and thus increased dead volume. The small size of the beta probe and lack of gamma sensitivity help to minimize these problems. Beta microprobe systems are also dual use, in that in addition to IF measurement they can be used in studies of the brain [12].

In those laboratories with access to a small animal PET scanner, the presently reported method allows the addition of IF with high temporal resolution at a relatively low cost, which could add value to these studies. For example, in combination with PET, the shunt allows the calculation of local cerebral glucose metabolism and kinetic rate constants in FDG studies using fully quantitative two-compartmental modeling approaches. In the case of studies into receptor occupancy and binding potential, the ability to measure IF not only allows the use of IF-reliant modeling (after the development of conversions for plasma and metabolism of the tracer) but also allows the comparison of binding data obtained using either IF or a reference region.

## Conclusion

In conclusion, the apparatus described in the present study allows the accurate determination of arterial input function in beta emitter radiotracer experiments in rats. It allows a high temporal resolution and minimizes blood loss; is ideally suited to radiotracer studies already utilizing light-tight beta microprobe systems as no extra counter is required; and is readily combined with PET in rats to add value to these studies.

## Competing interests

This research was supported by Johnson and Johnson Pharmaceutical Research and Development.

## Authors' contributions

GW drafted the manuscript, with editorial input from MB, AP, and AS. GW, MB, and AP carried out the beta microprobe and microPET studies. GW and MB performed the data analysis. DG, FG, CL, and JA performed and were responsible for FDG production. AS, AL, XL, and AP provided vital scientific input and edited the manuscript. All authors read and approved the final manuscript.

## Authors' information

This research was supported by FRS FNRS grant no. 3.4593.09 and by Johnson and Johnson Pharmaceutical Research and Development. AP is a senior research associate of the FRS-FNRS Belgium. MAB is supported by the FRS-FNRS Belgium ("Collaborateur logistique FRS-FNRS", grant 4.4508.08F).

## Supplementary Material

Additional file 1 **Supplementary Figure 1: Placement of the 2-mm VOI in the left ventricle of the rat heart image**. The myocardium is clearly shown in this average image from the last five time frames. In the lower right panel, the maximum-intensity projection image is shown (Supplementary Figure 1.doc, 190 K. http://www.ejnmmires.com/imedia/1534280600550379/supp1.doc). Click here for file

Additional file 2 **Supplementary Figure 2: Individual input functions derived using the three methods, corrected for injected activity and aligned for peak activity**. (**a**) probe-derived IFs; (**b**) manually sampled IFs; (**c**) image-derived IFs. (In order to maintain time-framing during alignment, data points are missing in some IFs) (Supplementary Figure 2.doc, 56 K. http://www.ejnmmires.com/imedia/1153689202550379/supp2.doc). Click here for file
