# Supplementary material for: Use of a beta microprobe system to measure arterial input function in PET via an arteriovenous shunt in rats
Source: EJNMMI Res. 2011 Aug 10;1:13. doi: 10.1186/2191-219X-1-13 (PMC3250971; doi:10.1186/2191-219X-1-13)

Supplementary Figure 1: Placement of the 2mm VOI in the left ventricle of the rat heart image. The myocardium is clearly shown in this average image from the last five time frames. In the lower right panel the maximum-intensity projection image is shown.


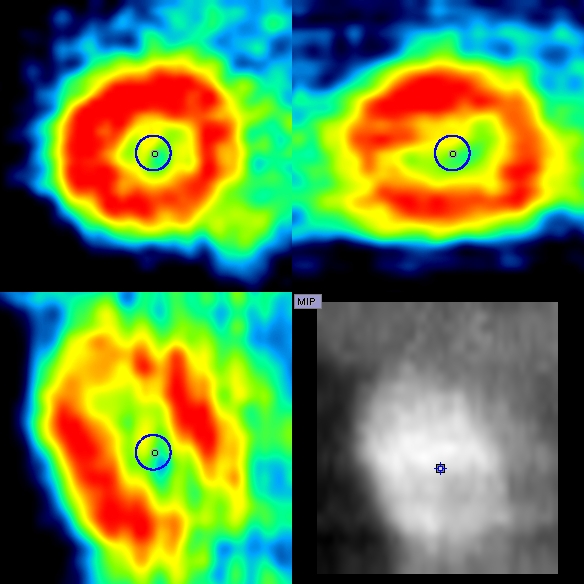

Supplement: Additional file 1 — Supplementary Figure 1: Placement of the 2-mm VOI in the left ventricle of the rat heart image. The myocardium is clearly shown in this average image from the last five time frames. In the lower right panel, the maximum-intensity projection image is shown (Supplementary Figure 1.doc, 190 K. http://www.ejnmmires.com/imedia/1534280600550379/supp1.doc). [file 2191-219X-1-13-S1.DOC]
