# Supplementary material for: Use of a beta microprobe system to measure arterial input function in PET via an arteriovenous shunt in rats
Source: EJNMMI Res. 2011 Aug 10;1:13. doi: 10.1186/2191-219X-1-13 (PMC3250971; doi:10.1186/2191-219X-1-13)
Supplement: Additional file 2 — Supplementary Figure 2: Individual input functions derived using the three methods, corrected for injected activity and aligned for peak activity. (a) probe-derived IFs; (b) manually sampled IFs; (c) image-derived IFs. (In order to maintain time-framing during alignment, data points are missing in some IFs) (Supplementary Figure 2.doc, 56 K. http://www.ejnmmires.com/imedia/1153689202550379/supp2.doc). [file 2191-219X-1-13-S2.DOC]

Supplementary Figure 2: Individual input functions derived using the three methods, corrected for injected activity and aligned for peak activity; a) probe-derived IFs; b) manually sampled IFs; c) image-derived IFs. (In order to maintain time-framing during alignment, data points are missing in some IFs).

a)

b)

c)
